# Exergaming System for Exercise-Based Cardiac Rehabilitation in Patients With Heart Failure: Development and Usability Assessment Study of a Device Prototype

**DOI:** 10.2196/71385

**Published:** 2025-07-16

**Authors:** Carles Blasco-Peris, Juan Pedro Alcolea Garrido, Barbara Seguí, Rocio Zaragoza, Vicente Climent-Paya, Laura Fuertes-Kenneally, Agustín Manresa-Rocamora, Ana Sanz-Rocher, Sabina Baladzhaeva, José M Sarabia

**Affiliations:** 1 Instituto de investigación sanitaria y biomédica de Alicante, Alicante, Spain; 2 Department of Physical Education and Sport, Universitat de València, Valencia, Spain; 3 Aiju, Ibi, Spain; 4 Department of Cardiology, Dr Balmis General University Hospital (HGUA), Instituto de investigación sanitaria y biomédica de Alicante, Av. Pintor Baeza, 12, Alicante, 03010, Spain, 34 965913941; 5 Department of Sport Sciences, Sports Research Centre, Universitat de Miguel Hernández d'Elx, Elche, Spain

**Keywords:** virtual reality, exercise, game-based rehabilitation, personalized intervention, HEFMOB, early mobilization, early rehabilitation

## Abstract

**Background:**

Heart failure (HF) is a growing global health concern, and adherence to early cardiac rehabilitation (CR) remains suboptimal. Exergaming is a promising alternative to conventional exercise programs for patients with HF. However, existing research has limitations, and the integration of exergaming into clinical practice remains challenging. Most notably, current studies often rely on commercially available systems that are not tailored to needs specific to patients with HF, lack long-term adherence strategies, and have limited evaluation in the initial phases of cardiac rehabilitation.

**Objective:**

This study aimed to design, develop, and assess the usability of a novel exergaming prototype (ie, HEFMOB), integrating immersive virtual reality (VR), real-time biometric monitoring, and autonomous session management to support early-phase, exercise-based CR in patients with HF.

**Methods:**

A multidisciplinary team developed HEFMOB through iterative prototyping. The final system included a pedal-based VR cycling game and an upper-limb mobilization minigame, with real-time monitoring of heart rate, blood pressure, and peripheral capillary oxygen saturation. Usability was assessed in two phases: (1) an expert evaluation and refinement phase and (2) a single-session usability phase involving 10 patients with HF (4 female). The sessions were recorded and individually evaluated by 2 researchers using the Serious Game Usability Evaluator tool. After each session, the participants completed the System Usability Scale (SUS) and a subscale of Intrinsic Motivation Inventory (IMI) to rate the usability of the exergaming prototype and enjoyment, respectively. Descriptive statistics were reported.

**Results:**

The participants had a mean age of 64.8 (SD 8.4) years, BMI of 26.7 (SD 4.6) kg/m^2^, and left ventricular ejection fraction of 40.5% (SD 7.4%). All participants completed the session without adverse events. The SUS score averaged 71.5, SD 17.8 (indicating good usability) and IMI scores indicated very high enjoyment (mean 25.1, SD 3.5). A total of 136 gameplay-related events were recorded: negative (n=76, mostly confusion), neutral (n=49), and positive (n=11). Interface-related issues (n=61) were most common, followed by design (n=52) and hardware (n=23).

**Conclusions:**

HEFMOB appears to be a promising, engaging, and well-tolerated tool for delivering tailored exergaming interventions in patients with HF. High usability and enjoyment ratings support its acceptability, while structured user experience analysis provided valuable insights for system refinement. This study marks a critical step toward integrating personalized, gamified exercise in inpatient settings, especially where early mobilization is lacking. Building on these findings, future research will assess long-term usability and clinical impact through a multicenter randomized controlled trial.

## Introduction

Heart failure (HF) is a growing global health concern in Europe and worldwide, leading to heavy economic costs, frequent hospitalizations, and high mortality [1]. Furthermore, trends suggest that prevalence and health care costs will continue increasing dramatically in the coming years. Despite significant advances in therapy and prevention, mortality and morbidity remain high, and quality of life is poor [2]. Hospitalization for HF is associated with a worse prognosis, reduced quality of life, and high costs [3]. Population aging, the rising prevalence of risk factors like hypertension, diabetes, obesity, and declining cardiovascular prevention and rehabilitation services all contribute to this alarming trend [4,5].

In this context, there is an acute need to optimize therapeutic strategies and administer treatments at the appropriate time. The World Health Organization has emphasized the need to strengthen cardiac rehabilitation (CR) within health care systems to meet the current and future needs of populations [6]. Within CR programs, an important aspect is phase 1 (ie, early mobilization), which is typically initiated within the first 24 to 72 hours of hospital admission once the patient is clinically stable [7]. Scientific evidence has shown that early multidomain physical rehabilitation intervention improves functional capacity and is associated with shorter hospital stays and fewer readmissions in patients with HF [8,9]. However, only 35% of the centers in Spain perform this phase 1 [10], mainly due to the absence of protocols and insufficient personnel, which hinder the application of these strategies. Thus, the low number of patients who do receive phase 1 CR is concerning, given that early CR may be their only opportunity for CR intervention [11]. The absence of financial incentives, space, multidisciplinary teams, and time are the most common barriers to implementing CR programs [12,13]. In light of these challenges, the accelerated pace of technological progress, particularly the convergence of internet of things (IoT) architecture and information and communication technologies, may offer a transformative avenue to address the mounting health care challenges [14-21]. Recent years have seen the emergence of exercise-based CR programs that implement the use of ICT, such as video games and virtual reality (VR), as alternatives to conventional exercise-based CR programs. These approaches represent a new way for patients to learn, participate in rehabilitation processes, receive personalized care, and engage in care interventions [16,22].

In addition to these advantages, immersive VR-based exergaming offers unique benefits: it enables patients to mentally escape the clinical environment, potentially reducing anxiety and enhancing psychological well-being; it has shown high acceptance and tolerance among vulnerable populations, such as older adults; and it effectively combines physical exercise with cognitive stimulation, which may improve mobility and cognitive function [23]. Exergaming has been demonstrated to be a safe and feasible intervention for patients with HF, although its effect on exercise capacity remains unproven in larger trials [24]. Pilot data suggest that exergaming may increase exercise capacity in selected patients [25].

Despite the promising results of exergaming studies, several crucial aspects and limitations have emerged [16,21]. First, the costs of exergaming equipment can be prohibitive [26]. Second, some patients may struggle with adopting new technology, and these commercial solutions may not be sufficiently tailored to individuals’ exercise needs [27]. Third, supervision and close monitoring may be necessary for specific patient groups and rehabilitation stages [28]. Finally, maintaining motivation to exercise can be a persistent challenge for patients, and exergaming alone may not be sufficient to ensure long-term adherence [29].

Having said that, few studies have addressed either the development, the testing, or both, of tailor-made exergaming systems in patients with cardiovascular disease [30-38]. Furthermore, there is a dearth of exergaming systems in the early phases of HF hospitalization that effectively tackle the limitations identified. Therefore, the aim of this study was to design, develop, and assess the usability of an exergaming prototype equipped with remote monitoring of biometric parameters and autonomous management functionalities. The prototype was intended to promote exercise-based CR in patients with HF.

Therefore, the aim of this study was to design, develop, and assess the usability of a tailor-made exergaming prototype for patients hospitalized with HF.

## Methods

### Study Phase 1: Development of the Exergaming Prototype

A multidisciplinary team of engineers, physicians, nurses, physiotherapists, and sports researchers designed and developed an exergaming prototype. The system underwent several development rounds to refine its functionalities and reach unanimous approval from all team members. The final prototype denominated the HF mobilization device (ie, HEFMOB), incorporates real-time monitoring of biometric variables and is specifically tailored for early mobilization in the HF setting.

**Figure 1.**
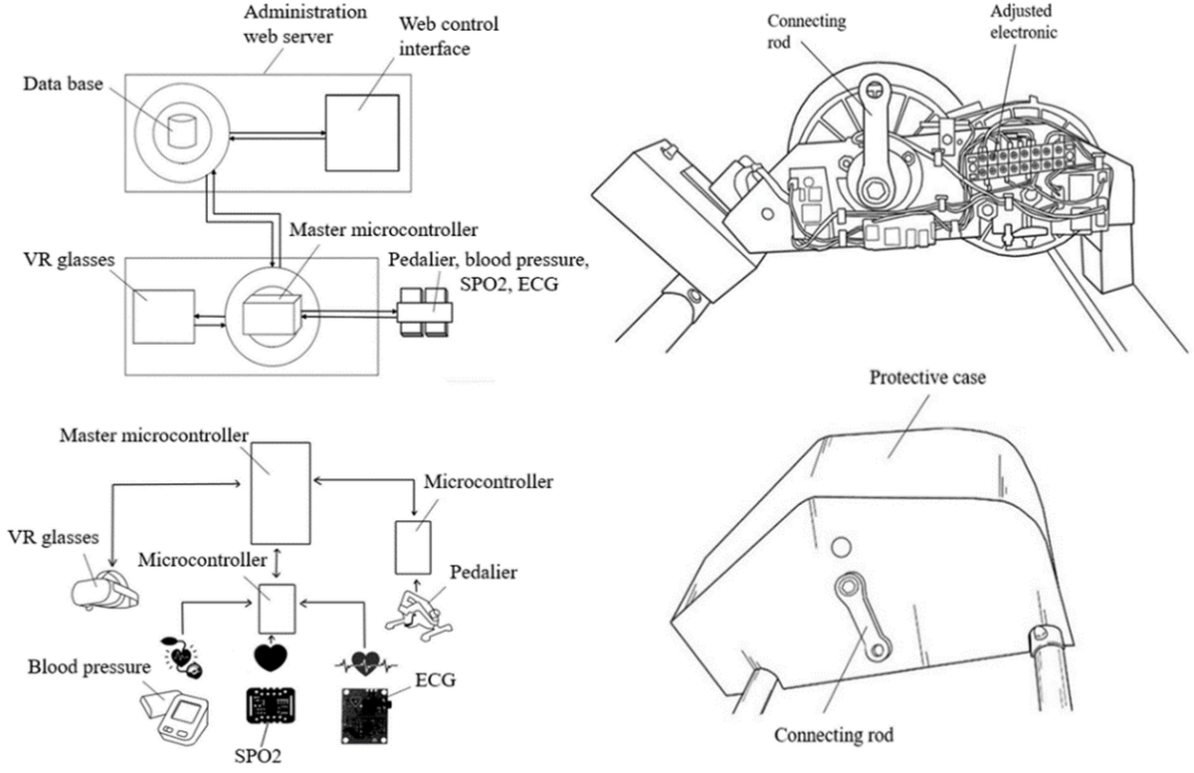
Prototype scheme diagram and electronic pedalier. VR: virtual reality; ECG: electrocardiogram; SpO_2_: peripheral capillary oxygen saturation.

HEFMOB is a therapeutic rehabilitation assistance device based on physical exercise and gamification. The device consists of the following essential elements: microcontrollers, biometric data measurement devices, an administration web server with a web control interface, data communication technologies, a pedalier, and VR glasses (see Figure 1). The Spanish Agency of Medicines and Health Products approved the prototype to conduct clinical studies as a class IIA clinical product (974/22/EC-R). In addition, its intellectual property rights have been registered with the Spanish Patent and Trademark Office (U202330170).

The centerpiece of HEFMOB is the integration of a VR videogame tailored to the Oculus Quest 2 (Meta Platforms), a cutting-edge VR platform renowned for its immersive experiences [39]. The game is integrated into an IoT architecture orchestrated by Raspberry Pi and supported by ESP32 devices that transmit critical physiological parameters [40], such as peripheral capillary oxygen saturation (SpO_2_), blood pressure, and heart rate, in real-time through the Message Queuing Telemetry Transport protocol [41].

**Figure 2.**
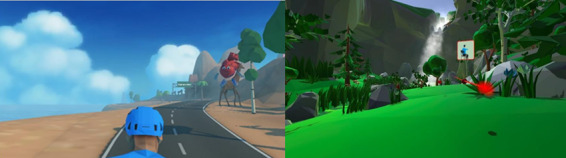
Virtual reality–based road cycling tour and fruit-picking minigame.

All collected data are stored in a database and displayed in a dashboard, allowing health care professionals to monitor real-time patient metrics through a web-based control interface during each session. Users engage with the VR game by pedaling through various scenarios while wearing sensors that measure heart rate, SpO_2_, blood pressure, and electrical heart activity. An avatar named Cori guides patients throughout the game, offering instructions and information on how to interact with the system autonomously (see Figure 2 left). Pedal resistance is automatically adjusted based on the target heart rate set by the specialist. This target is established by the clinical team according to guidelines for inpatient cardiac rehabilitation in the absence of a stress test and is set approximately 30 beats per minute above the patient’s resting heart rate before the session [42]. The system continuously monitors heart rate in real time and adjusts pedaling resistance every 5 seconds to maintain it within ±2 bpm of a target. Based on the average of the last 5 readings, resistance increases or decreases by one unit (range 0‐8) to match cardiovascular demand, ensuring a safe and adaptive workload. Participants pedal alongside a virtual partner and compete against an opponent. To add excitement, patients collect water bottles to prevent their partner from dehydration. Along the route, participants encounter billboards integrated into the virtual scenario with information relevant to HF pathology. This information, in turn, aims to provide patients with tips on managing their condition better and promote health-conscious behaviors. Once the route phase is over, users engage in a fruit-picking minigame (see Figure 2 right). In this segment, fruits are directed toward the patients, prompting them to carry out upper limb movements corresponding to the fruits’ trajectories. The difficulty of the game depends on the speed and frequency at which patients are required to pick the fruit.

Following the minigame, the Borg CR-10 scale [42,43] is displayed, and patients are asked to assess their rate of perceived exertion (RPE) after completing both VR segments. These data are also collected by the control system.

### Study Phase 2: Usability Assessment

Patients aged 50 to 74 years, diagnosed with HF with mildly reduced (40% to 50%) or reduced (<40%) left ventricular ejection fraction, and without cognitive or physical limitations, were recruited from the HF Unit at Dr Balmis General University Hospital of Alicante. All participants provided written informed consent before being included in the study. Exclusion criteria were unstable angina pectoris, decompensated HF, myocardial infarction in the last 6 months, diabetes mellitus, chronic obstructive pulmonary disease, and complex ventricular arrhythmias. The usability of the prototype was studied in stable patients with HF (ie, chronic heart failure [CHF]) to confirm its safety before using it with decompensated HF. VR experiences were asked before starting usability sessions.

The usability assessment was performed according to evidence-based methods [44], in two stages: (1) expert methods and (2) user methods (ie, patients with CHF). In the first stage, 7 research team members (2 cardiologists, 2 nurses, 2 sport science graduates, and 1 engineer) engaged with the prototype themselves. Based on their insights, necessary modifications to the prototype’s functionality were made to improve its usability.

Subsequently, the usability assessment with patients took place. A total of 10 patients with CHF (6 males and 4 females) used the prototype for approximately 20 minutes in a single session in a simulator clinical laboratory. Every session started by placing the hardware elements on the participants. Participants were asked to follow Cori’s instructions, while researchers refrained from giving feedback or suggestions during the sessions. Later, Cori provided a brief introduction with a functional explanation and 1 example for familiarization purposes. Thereafter, the cycling tour started, and all patients performed a single 5-minute bout of pedaling at 60% to 70% of their predicted maximum heart rate followed by the fruit-picking minigame, as previously discussed in study phase 1. The session concluded with a question regarding their RPE.

**Figure 3.**
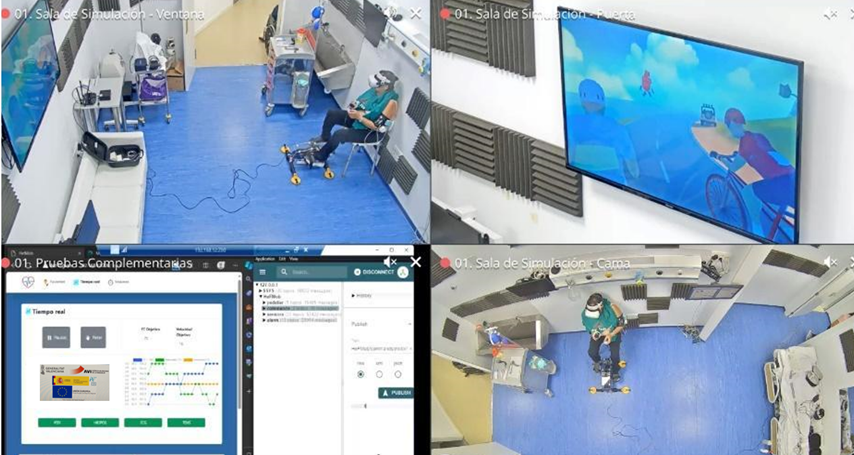
Example of a recorded participant session.

Furthermore, 2 methodological approaches were used to assess familiarization and usability. First, observational analyses were performed while the users interacted with the system. During this stage, patients were instructed to verbalize their thoughts and actions while their sessions were recorded via video and audio using the CAE Learning Space platform (see Figure 3). Subsequently, metrics of game familiarization (ie, percentage of bottles acquired, average time to pick each of the first 5 bottles, and percentage of fruits acquired) were exported from the system database. In addition, 2 research team members independently reviewed the recorded sessions and documented gameplay and usability-related events using the Serious Game Usability Evaluator (SGUE) tool [45].

The SGUE is a structured method designed to evaluate a serious game, focusing not only on the prototype itself but also on the process of interacting with the game and the user’s experience. Events recorded with the SGUE are categorized based on their nature (interface, design, and hardware) and the user’s reaction (negative, neutral, or positive). This comprehensive approach provides a deeper understanding of how users interact with the game, highlighting areas for improvement in both functionality and user satisfaction [45]. Because the HEFMOB prototype comprises various gadgets, the evaluation also encompassed the interaction with hardware elements. Any disagreements between the two assessors were resolved through consensus. A list of action points to improve system usability was derived from the analysis of the SGUE results. For each action related to a neutral or negative user dimension, we indicated the frequency (number of events that would be solved by this action) and the dispersion (number of users that encountered an event that would be solved by this action). Both numbers were used to determine the priority of each action.

Survey-based methods were also used. Immediately after the session, patients were asked to answer the System Usability Scale (SUS) [46], a 10-item Likert scale with 5 possible levels of agreement, ranging from strongly disagree to strongly agree. The SUS score was interpreted as worst imaginable (≥12.5), awful (≥20.3), poor (≥35.7), ok (≥50.9), good (≥71.4), excellent (≥85.5), and best imaginable (≥90.9) [47]. Finally, patients also responded to the 5-item interest and enjoyment subscale of the Intrinsic Motivation Inventory (IMI) [48]. The IMI score was interpreted as follows: very low level of enjoyment (5‐9 points), low level of enjoyment (10‐14 points), moderate level of enjoyment (15‐19 points), high level of enjoyment (20 to 24 points), and very high level of enjoyment (25 to 35 points) [49]. Mean and SD values are reported.

### Ethical Considerations

This study was conducted in accordance with the principles outlined in the Declaration of Helsinki and received approval from the Comité Ético de Investigación con Medicamentos del Departamento de Salud de Alicante – Hospital General (2022/041). All participants provided written informed consent before inclusion. The approved consent form included explicit provisions for the analysis and secondary use of the collected data. To protect privacy and confidentiality, all data were deidentified before analysis, and no personal or identifying information was stored or shared. Participation was entirely voluntary, and no compensation was offered or received. In addition, no individual participant can be identified in any of the images or multimedia appendices included in the manuscript.

## Results

### Study Phase 2: Usability Assessment

#### Expert Methods

At this stage, areas for improvement were identified and subsequently addressed (see Multimedia Appendix 1 for the list of needs and improvements in the experts’ methods).

Key modifications included adding suction cups to the pedaling mechanism for stability and automating pedaling intensity based on the target heart rate, calculated from the average of the previous 5 heart rate values. This removed the need for a manual intensity test. Usability improvements included removing the password requirement to start sessions, adding portable batteries for the VR glasses, and enabling real-time monitoring and archiving of blood pressure and SpO_2_ data, with the option to manually measure blood pressure during adverse events. Minigame updates introduced gameplay explanations, difficulty levels, replay options, and adjustments to reduce dizziness by optimizing collider areas. Additional improvements included redesigning the pulse oximeter holder for consistent signals, updating blood pressure measurement methods to support the arm, and adding an easily accessible emergency stop button, both physically and via the web interface. These changes enhanced the system’s functionality, safety, and user experience.

These updates improved the functionality and expert user experience of the system.

#### User Methods

Table 1 shows the characteristics of the 10 participants with CHF who completed the session. None of the recruited patients had any previous experience with VR.

**Table 1.** Participant characteristics (n=10).

Variable		Values
Quantitative variables, mean (SD)		
Age (years)		64.8 (8.4)
Weight (kg)		72.3 (14.1)
Height (cm)		164 (0.1)
BMI (kg/m^2^)		26.7 (4.6)
LVEF^a^ (%)		40.5 (7.4)
Categorical variables, n (%)		
LVEF		
Mildly reduced		6 (60)
Reduced		4 (40)
Aetiology		
Ischemic		3 (30)
Nonischemic		7 (70)
Sex		
Male		6 (60)
Female		4 (40)
VR^b^ experience		
Yes		0 (0)
No		10 (100)

a LVEF: left ventricular ejection fraction.

b VR: virtual reality.

Regarding familiarization, participants successfully acquired 72 out of 112 bottles during the tandem cycling tour and 179 out of 590 fruits in the fruit-picking minigame. The average time participants took to acquire each of the first 5 bottles show a downward trend along the session (see Figure S1 in Multimedia Appendix 1).

Table 2 shows the number of appearances of each tag and the frequencies for each event type obtained with the SGUE tool. More negative events (76/136, 56%), such as confusion, than neutral (49/136, 36%) and positive events (11/136, 8%) were recorded. In addition, there were more interface events (61/136, 45%) than events related to design (52/136, 38%) and hardware (23/136, 17%).

Table 2 summarizes the number of appearances of each tag and the frequencies for different event types, as recorded by the SGUE tool (the dataset is available at [50]). A total of 136 events were categorized. Negative events (n=76, 56%) were the most prevalent, with confusion being the dominant tag (n=48), particularly in interface (n=23) and design-related issues (n=11). For example, users frequently expressed confusion when navigating certain interface features or attempting to understand design elements like layout inconsistencies.

**Table 2.** Tag statistics: the events are categorized into 2 dimensions: the source of the event (interface, design, and hardware) and the reaction of the user.

User-related dimension	System-related dimension							
	Interface, n (%)				Design, n (%)		Hardware, n (%)	Total, n (%)
	Content	Layout and UI		Technical error	Gameflow	Functionality		
Negative								
Annoyed	0 (0)	0 (0)		0 (0)	0 (0)	0 (0)	0 (0)	0 (0)
Confused	7 (5)	23 (17)		0 (0)	6 (4)	11 (8)	1 (0)	48 (35)
Frustrated	0 (0)	1 (0)		0 (0)	3 (2)	3 (2)	0 (0)	7 (5)
Unable to continue (fatal)	0 (0)	0 (0)		0 (0)	0 (0)	6 (4)	15 (11)	21 (15)
Positive								
Pleasantly frustrated	0 (0)	0 (0)		0 (0)	2 (1)	0 (0)	0 (0)	2 (1)
Reflecting	0 (0)	1 (0)		0 (0)	0 (0)	0 (0)	0 (0)	1 (0)
Satisfied or excited	0 (0)	0 (0)		0 (0)	1 (0)	0 (0)	0 (0)	1 (0)
Learning	0 (0)	1 (0)		0 (0)	4 (3)	2 (1)	0 (0)	7 (5)
Neutral								
N/A^a^	2 (1)	1 (0)		21 (15)	0 (0)	3 (2)	6 (4)	33 (24)
Suggestion or comment	1 (0)	3 (2)		0 (0)	9 (7)	2 (1)	1 (0)	16 (12)
Total	10 (7)	30 (22)		21 (15)	25 (18)	27 (20)	23 (17)	136 (100)

a N/A: not available.

Neutral events (n=49, 36%) primarily involved suggestions or comments (n=16) and technical observations that did not provoke strong reactions, such as noting specific interface layouts or reporting minor glitches.

Positive events (n=11, 8%) were less common, with users occasionally reporting satisfaction (n=2) or instances of learning (n=7). A practical example is a user adapting to a new feature after initial difficulty, ultimately finding the experience rewarding.

From a system-related perspective, interface issues accounted for the largest share of events (n=61, 45%), followed by design-related problems (n=52, 38%) and hardware issues (n=23, 17%). Specific examples of interface challenges included unclear menu structures or misaligned buttons. Hardware-related events, though less frequent, were impactful, such as issues with the positioning or adjustment of sensors, which sometimes affected gameplay.

A list of action points to improve system usability was derived from the analysis of the results in Table 2 (see Table 3). The interface action points provided can be grouped into graphics issues (priority 1) and comprehension issues with the avatar (priorities 5 to 7). The hardware action points shown can be categorized into hardware elements that users interact with (priorities 2 and 8) and hardware elements for user monitoring (priority 9). The design action points presented can be classified as user imprecise execution (priorities 3 and 4).

Table 4 displays the results of the usability assessment. Just 1 participant experienced a light dizziness sensation along the curves in the road cycling tour. All participants expressed interest and enjoyment after the prototype session. The participants who took part in the usability assessment reported very high levels of interest/enjoyment (mean 25.1, SD 3.5) and rated the exergame’s usability as good (mean 71.5, SD 17.8).

**Table 3.** Excerpt of the prioritized action points list.

Priority	Action	Type	Frequency	Spread
1	Error with an image.	Hardware	20	10
2	Assistance to deliver the second controller.	Interface	11	10
3	Difficulty activating the command.	Design	14	9
4	Technical problem performing the action.	Design	15	8
5	User does not understand Cori’s instruction.	Interface	15	8
6	Question regarding Cori’s instruction.	Interface	9	6
7	Lack of indication from Cori.	Interface	10	5
8	Issues with the pedal anchor.	Hardware	9	5
9	Maladjustment of one of the devices gathering hemodynamic data.	Hardware	7	4

**Table 4.** Usability assessment outcomes.

Participant ID	Dizziness	IMI^a^ score	IMI level of interest/enjoyment	SUS^b^ score	SUS usability
1	No	24	High	37.5	Poor
2	No	19	Moderate	75	Good
3	No	28	Very high	97.5	Best imaginable
4	No	28	Very high	70	Ok
5	No	25	Very high	45	Poor
6	No	19	Moderate	82.5	Good
7	No	27	Very high	77.5	Good
8	No	28	Very high	75	Good
9	Yes	28	Very high	82.5	Good
10	No	25	Very high	72.5	Good
Mean (SD)	—^c^	25.1 (3.5)	Very high	71.5 (17.8)	Good

a IMI: Intrinsic Motivation Inventory.

b SUS: System Usability Scale.

c Not applicable.

Mean RPE was 4.5 (SD 3.0; see Table S1 in Multimedia Appendix 1). This indicates a moderate perceived effort during the session, with individual values ranging from 0 to 8, reflecting variability in the participants’ exertion levels.

## Discussion

### Principal Findings

This study presents the design, development, and usability evaluation of HEFMOB, an exergaming prototype integrating remote monitoring and autonomous management features to support early-phase, exercise-based CR in patients with HF. The prototype was developed through a multidisciplinary collaboration with technological and health research institutes, hospitals, and academia and tested in a sample of 10 patients, all without previous VR experience. Overall, the usability assessment showed that the system was safe, well tolerated, and highly engaging. Patients reported high levels of interest and enjoyment (IMI mean 25.1, SD 3.5) and rated the system’s usability as good (SUS mean 71.5, SD 17.8), with only 1 patient reporting a light sensation of dizziness during a specific activity, which was not classified as an adverse event. Although some negative user feedback was recorded, primarily related to confusion with interface and game design, the system was perceived positively overall. A systematic analysis of 136 user experience events led to a prioritized list of actionable improvements, which have informed the next version of the prototype. These findings support the feasibility and user acceptability of HEFMOB as a novel solution for implementing early mobilization in hospitalized patients with HF. The analysis revealed a notable prevalence of negative events (n=76) compared to neutral (n=49) and positive ones (n=11), with interface issues (n=61) outweighing design (n=52) and hardware (n=23) concerns. These results are similar to another Serious Game for patients [44], with negative events being the most common, representing approximately 50%, followed by neutral events, and finally, positive events. In software development, it is usual that some functions are obvious for developers and experts but not for real users, leading to negative events such as feelings of confusion or frustration [51].

Action points were formulated based on the events analysis findings, with prioritization determined by the frequency of occurrence and the number of users affected. Addressing interface issues related to graphics emerged as the top action point. Other hardware and design actions were identified to improve interaction with physical elements and enhance game execution. These improvements will be incorporated into the next HEFMOB prototype version.

In the usability assessment, participants largely avoided dizziness, with only 1 participant reporting a mild dizziness sensation during a specific activity. Overall, participants expressed significant interest and enjoyment, rating the exergame’s usability positively. Despite the good scores on the SUS, 2 participants rated usability poorly and provided comments about disliking the bicycle exercise and finding the fruit-picking game difficult. Taking into consideration their scores on the IMI (both rated the activity 26, a very high level of enjoyment), we hypothesize that the 2 participants who rated the system usability as poor were influenced by their dislike of exercise and the difficulty of the game rather than system functioning or usability. This could explain the poor usability score. Although patients received a brief introduction and familiarization with the system before each exercise session, age-related factors should also be taken into account. Age and previous gaming experience can influence usability and the occurrence of negative events. Older adults may struggle with digital literacy due to the digital divide, limiting effective interaction with active video games [20]. Proper onboarding is therefore essential to address these challenges, reinforcing the need for tailored familiarization based on users’ age and gaming experience [20].

While this feasibility study and its findings are exploratory in nature, they nonetheless offer valuable insights. In a sample of 10 patients with HF, the HEFMOB prototype demonstrated no side effects, good usability, and user enjoyment. Furthermore, in line with Kortum and Peres [52], who established a correlation between SUS scores and satisfaction, the usability assessment outcomes suggest that patients with HF experienced overall satisfaction after a single session with the HEFMOB prototype.

### Comparison With Previous Work

Other studies have developed exergaming prototypes for exercise-based CR [30-34,36,38], but few have been used in patient samples that included children and adolescents with congenital heart disease or in patients undergoing nonemergency coronary artery graft surgery [32,35,37]. Among these studies, 2 different exergaming systems stand out: an augmented reality software developed to train patients in physical activities, such as walking around the inpatient ward and climbing stairs [32]; and the MedBike, an innovative rehabilitation home-based device designed to enhance patient adherence, convenience, and engagement [35,37]. MedBike has a 2-tiered interface, enabling clinicians to remotely monitor patients as they exercise on a stationary bicycle while immersed in a VR environment. Advanced sensors continuously track vital signs (ie, electrocardiograph, SpO_2_, and blood pressure), providing valuable data for personalized care [30].

HEFMOB and MedBike differ in that HEFMOB has been designed and developed for use in the early phase, in a hospital setting, whereas MedBike is used in an outpatient setting. HEFMOB can be used with any conventional chair, whereas MedBike requires the use of a cycle ergometer. HEFMOB uses immersive VR technology, whereas MedBike does not. In addition, HEFMOB boasts unique functionalities such as autonomous management of sessions guided by an avatar, autonomous regulation of pedaling intensity based on the predetermined target heart rate, inclusion of upper body mobilization exercises, promotion of healthy habits, and management of pathology through in-game advice.

Our results suggest that HEFMOB represents a significant initial step in developing an exergaming prototype customized for patients with HF. It encourages exercise-based CR in a patient population while also incorporating remote biometric monitoring and autonomous management. Based on the findings from this pilot study, we believe that the HEFMOB prototype holds promise for promoting early exercise-based CR at hospital facilities. Its implementation may facilitate the attainment of desirable engagement levels in phase 1 CR programs and compliance with early mobility recommendations in line with clinical guidelines [3,53]. Furthermore, the impact of HEFMOB could be notably amplified given that the early CR phase may be the sole opportunity for CR intervention in a substantial number of patients [11].

### Limitations and Research Priorities

The study involved only 10 patients with stable CHF, which may limit the generalizability of the findings to HF inpatients, particularly those who have experienced recent decompensation. A larger sample size would provide more robust evidence regarding the usability of HEFMOB across a broader patient population. In addition, the usability assessment was based on a single session with HEFMOB, which may not fully capture its long-term usability. This phase was necessary before applying HEFMOB in real-world scenarios with patients post decompensation, which will be addressed in subsequent studies. Furthermore, future studies will also evaluate the effectiveness of HEFMOB in improving patient outcomes.

The assessment of usability relied primarily on subjective measures, such as participants' ratings and feedback. While these provide valuable insights, future studies could consider complementing subjective data with additional measures, such as detailed session observations, to provide a more comprehensive evaluation of HEFMOB’s usability. Importantly, this study did not aim to analyze effectiveness, and metrics related to performance or physiological responses were not included, as these will be explored in the planned effectiveness studies.

### Conclusions

The findings suggest that HEFMOB is a feasible and well-tolerated tool, demonstrating high user engagement and good usability ratings in a controlled, single-session simulation clinical laboratory environment involving patients with HF in the ambulatory phase. HEFMOB represents a significant initial step in the development of an exergaming prototype tailored to the needs of patients with HF and emerges as a promising and engaging tool for supporting early exercise-based CR.

### Future Work

Future research is required to conclusively demonstrate the efficacy and effectiveness of serious exergaming prototypes such as HEFMOB in promoting early mobilization in hospital, particularly through interventions involving multiple sessions. These future analyses should also examine its subsequent impact on duration of hospital stay, readmissions, and behavioral and lifestyle outcomes, including quality of life, improved disease knowledge, functional capacity, and engagement and adherence in physical activity and exercise.

Next steps include the deployment of a new prototype version of the system. A new usability study is planned with a larger sample, multiple sessions, and semistructured interviews with participants who may report poor usability. This approach aims to generate deeper insights into user experience and system performance, guiding further refinements before scaling.

Subsequently, a multicenter randomized controlled trial will be conducted, with sample size calculated based on expected effect sizes. The study will assess outcomes such as functional capacity, muscular strength, HF knowledge, body composition, psychosocial status (anxiety and depression), and user enjoyment. Gender representation will be monitored to address potential biases, and rational exclusion criteria (eg, cognitive impairment, severe comorbidities, and low digital literacy) will ensure participant safety and data quality. Follow-up will include hospital readmissions and all-cause mortality to evaluate the long-term clinical impact of the intervention.

## Supplementary material

10.2196/71385 Multimedia Appendix 1 Expert-identified needs and proposed improvements, individual game outcomes with perceived exertion ratings, and average time to task completion.

## Abbreviations list

CHF: chronic heart failure
CR: cardiac rehabilitation
HF: heart failure
IMI: intrinsic motivation inventory
IoT: internet of things
RPE: rate of perceived exertion
SGUE: Serious Game Usability Evaluator
SpO_2_: peripheral capillary oxygen saturation
SUS: System Usability Scale
VR: virtual reality
