# Supplementary material for: Exergaming System for Exercise-Based Cardiac Rehabilitation in Patients With Heart Failure: Development and Usability Assessment Study of a Device Prototype
Source: JMIR Serious Games. 2025 Jul 16;13:e71385. doi: 10.2196/71385 (PMC12286565; doi:10.2196/71385)
Supplement: Multimedia Appendix 1 [file games-v13-e71385-s001.doc]

## MULTIMEDIA APPENDIX 1. Expert-identified needs and proposed improvements, individual game outcomes with perceived exertion ratings, and average time to task completion.

### List of need and improvements in the experts’ methods

- Changes to the pedalling mechanism: addition of suction cups.
- Removal of the password requirement to start a session with the Oculus Rift.
- Exportation of data in CSV format.
- Adjustment of the pedalling intensity based on the target heart rate, and elimination of the pedalling intensity test performed by the patient.
- Intensity is now automatically regulated based on the target heart rate and actual heart rate (average of the previous five values, with a margin of error of ±2 beats per minute).
- Increase in the number of pedalling intensities.
- Incorporation of contralateral upper limb movements into the mini-game.
- Change to the points of passage of the circuit (collider areas) to minimize dizziness: increase in the number, decrease in the area, and compaction of colliders.
- Mini-game modifications: elimination of warm-up, introduction of various difficulty levels, and option to replay.
- Prevention of bottles from appearing during blood pressure recording and centering of their appearance during the course.
- Increased time for detecting pedalling and synchronization with the “not pedalling” message.
- Modification of the blood pressure image: the arm must be supported, not suspended.
- Revised method to select the level of effort for the session. Now, the level of effort must be marked on the image with the controller, by pressing the controller button.
- Emergency stop button is now readily accessible for user, with activation available through the web control interface.
- Introduction of an explanation of the mini-game indicating that the position to catch the fruits must be maintained until the fruits explode. In an updated version, Cori explains that the position must be maintained until the fruit explodes.
- Real-time display and archiving of blood pressure and SpO_2_.
- Change to the design of the pulse oximeter holder to obtain a constant signal without loss.
- Incorporation of the function to manually measure blood pressure outside of the game (in an adverse event, e.g., dizziness).
- Implementation of portable batteries for VR glasses.
- Improvement of the ECG signal.

### Table supplementary material S1. Individual game outcomes and rate of perceived exertion (RPE).

| **Participant ID** | **RPE** |  | **Bottles** | | |  | **Fruits** | | |
| --- | --- | --- | --- | --- | --- | --- | --- | --- | --- |
|  |  |  | **N** | **Total** | **%** |  | **N** | **Total** | **%** |
| 1 | 5 |  | 8 | 12 | 67 |  | 14 | 59 | 24 |
| 2 | 2 |  | 0 | 10 | 0 |  | 8 | 59 | 14 |
| 3 | 6 |  | 10 | 10 | 100 |  | 19 | 59 | 32 |
| 4 | 2 |  | 11 | 11 | 100 |  | 27 | 59 | 46 |
| 5 | 7 |  | 0 | 11 | 0 |  | 12 | 59 | 20 |
| 6 | 7 |  | 10 | 13 | 77 |  | 4 | 59 | 7 |
| 7 | 1 |  | 12 | 12 | 100 |  | 31 | 59 | 53 |
| 8 | 8 |  | 0 | 11 | 0 |  | 13 | 59 | 22 |
| 9 | 7 |  | 10 | 11 | 91 |  | 28 | 59 | 47 |
| 10 | 0 |  | 11 | 11 | 100 |  | 23 | 59 | 39 |
| **Mean (SD)** | **4.5 (3.0)** |  | **7.2 (5.1)** | **11.2 (0.9)** | **63.5 (45.2)** |  | **22.7 (21.3)** | **64.9 (18.7)** | **31.8 (18.1)** |

SD: standard deviation

### Figure S1. Average time taken to acquire bottles


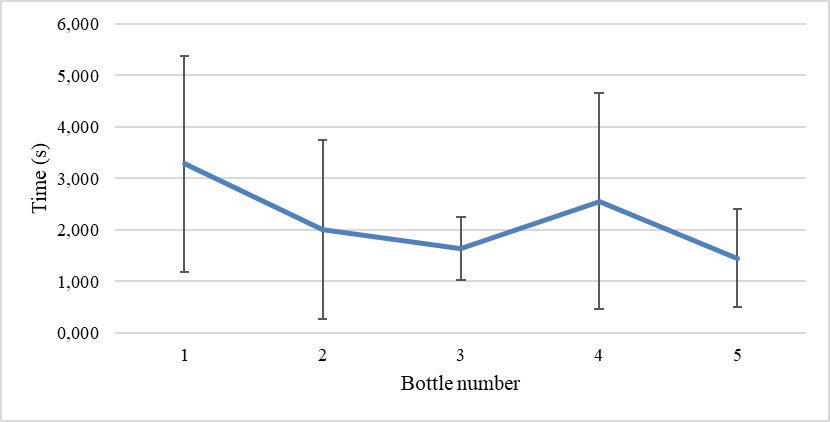


The dataset presented in Table S1 and Figure S1 captures user interaction and object acquisition performance during two gamified activities integrated into a cardiac rehabilitation system and can be accessed at the following link: <https://zenodo.org/records/15323173>
